# Therapeutic Potential of Perillaldehyde in Ameliorating Vulvovaginal Candidiasis by Reducing Vaginal Oxidative Stress and Apoptosis

**DOI:** 10.3390/antiox11020178

**Published:** 2022-01-18

**Authors:** Lei Chen, Fei Wang, Su Qu, Xiaona He, Yongxin Zhu, Yi Zhou, Kunlong Yang, Yong-Xin Li, Man Liu, Xue Peng, Jun Tian

**Affiliations:** School of Life Science, Jiangsu Normal University, Xuzhou 221116, China; chenlei_2228@163.com (L.C.); 3020172463@jsnu.edu.cn (F.W.); qusu_1111@163.com (S.Q.); xiaona_he_n@163.com (X.H.); 3020172883@jsnu.edu.cn (Y.Z.); 3020171423@jsnu.edu.cn (Y.Z.); ykl_long@jsnu.edu.cn (K.Y.); yongxinli@jsnu.edu.cn (Y.-X.L.); liuman@jsnu.edu.cn (M.L.); pengxue@jsnu.edu.cn (X.P.)

**Keywords:** *Candida albicans*, perillaldehyde, vulvovaginal candidiasis, ROS, antioxidation, natural products, antimicrobial

## Abstract

Vulvovaginal candidiasis (VVC) is one of the most frequent diseases induced by *Candida albicans* (*C. albicans*) during pregnancy, which results in enormous pain to women and their partners in daily life. Perillaldehyde (PAE), a natural monoterpenoid, has significant anti-microbial, anti-inflammatory and anti-oxidation effects. Reactive oxygen species (ROS) are key factors for the host to resist the invasion of fungi. However, excess ROS can cause additional damage independent of the pathogen itself, and the mechanism of ROS in VVC has not been investigated. In this murine study, we revealed that *C. albicans* infection increased the expression of NADPH oxidase 2 (NOX2) and the content of malonaldehyde (MDA). *C. albicans* inhibited the activity of antioxidant enzymes in the vagina, including superoxide dismutase (SOD), Catalase (CAT), glutathione peroxidase (GSH-PX) and heme oxygenase (HO-1), which were returned to normal levels after treatment with PAE. Furthermore, PAE inhibited the activities of Keap1 and promoted Nrf2 transfer from cytoplasm to nucleus, which were mediated by excessive accumulation of ROS in the VVC mice. In this study, we also indicated that PAE inhibited the apoptosis of vagina cells via Caspase 9- Caspase 7-PARP pathway and prevented the release of IL-1ꞵ in VVC mice. In summary, this study revealed that the treatment of VVC in mice with PAE might be mediated by inhibition of ROS, and established the therapeutic potential of PAE as an antifungal agent for the treatment of VVC.

## 1. Introduction

Vulvovaginal candidiasis (VVC) is a pervasive disease caused by a symbiotic fungus *Candida albicans* (*C. albicans*), which has been previously shown to be parasitic on the mucosal surface. VVC is often accompanied by pain, itching, burning, redness of the vulva and a large amount of vaginal discharge [1]. The use of high-estrogen oral contraceptive, hormone replacement therapy, misuse of antibiotic, and the emergence of a large number of diabetic female patients, have contributed to the increasing prevalence and recurrence of VVC [2]. In fact, about 75% of women are afflicted by VVC at least once in their life time, especially during the childbearing age and menstrual cycle when their estrogen level is elevated. Estimated suggest that 5–8% of women (about 150 million all over the world) suffer from recurrent invasion of *C. albicans*, which effectuates great affliction to their daily life, as well as causing great challenges to their clinical treatment [3]. Currently, available antifungal agents have a variety of shortcomings and due to the adept mutation of *C. albicans*, a variety of clinical fungal resistance have been discovered. Amongst the numerous mainstream oral antifungal agents for the treatment of VVC, fluconazole has displayed the most favorable antifungal activity spectrum with favorable pharmacokinetics. However, the resistance of *C. albicans* to azole has now been detected in women with VVC, and the long-term treatment of VVC still remains a bottleneck task to accomplish [4,5]. Therefore, it is urgent to develop novel antifungal agents.

Considerable evidence has indicated that poor prognosis and recurrent symptomatic infections result from the overreaction of the immune system towards pathogenic fungi [6,7]. Cells of the innate immune system secrete a large amount of reactive oxygen species (ROS) useful in combating pathogenic microorganisms upon stimulation. Interestingly, ROS can function as a sensor to enable the immune system to react proportionally to microorganism invasion [8]. Therefore, ROS play a crucial role in the defense of microbial infection. Presently, the development of antifungal drugs is shifting towards natural products [9,10]. *Perilla frutescens* is an important annual herb of the mint family. Widely distributed in East Asia, especially China, Korea and Japan, it is broadly used as a fresh vegetable and culinary herb [11]. Our previous study indicated that perillaldehyde (PAE), which is a natural monoterpene extracted from *Perilla frutescens*, has a promising antimicrobial effect [12,13,14,15]. PAE has been reported to have antibacterial effects, such as the growth inhibition of *Micrococcus luteus*, *Staphylococcus epidermidis*, *Bacillus cereus*, and *Corynebacterium sp.* [16]. In addition, PAE has also been reported to have some antifungal effects and can inhibit the growth of *Aspergillus flavus*, *Aspergillus oryzae*, *Aspergillus niger*, *Alternaria alternate*, *Ceratocystis fimbriata* and *Penicillium sp*. [15,17,18,19]. Furthermore, PAE possesses an anti-parasitic effect as shown in the growth inhibition of *Leishmania donovani* [20]. PAE has been certified as generally safe by the expert group of the American Association of Flavorings and Extracts Manufacturers (FEMA), the Food and Agriculture Organization of the United Nations (FAO), World Health Organization (WHO) and the Joint Expert Committee on Food Additives (JECFA) (2003) [21]. Furthermore, previous studies have reported that PAE possesses outstanding anti-depressant, anti-inflammatory and antioxidant effects [22,23,24]. Our previous studies indicated that PAE inhibited the growth of *C. albicans*, biofilm formation, the transformation of mycelium phase and the expression of secreted aspartic proteinases (SAPs) [14]. At the same time PAE, as a natural terpenoid compound with better absorption and excretion capabilities, has been reported to possess the ability to target the virulence protein of *C. albicans* [14,25]. In addition, our previous results have shown that PAE could reduce cytokine levels and immune cell content in the vagina of VVC mice [13]. Unfortunately, the mechanism of PAE in ameliorating VVC remains unclear. Considering that ROS are important factors that activate NLRP3 inflammasome and triggers a host inflammatory response [26], this study aims to further clarify the mechanism of PAE in ameliorating VVC, and provides evidence that is useful for the development of PAE as a novel antifungal agent.

## 2. Materials and Methods

### 2.1. Main Reagents

Perillaldehyde (CAS registry no. 18031-40-8) was purchased from the TCI Development Co., Ltd. (Tokyo, Japan), prepared as a stock solution in 0.1% (*v*/*v*) Tween-80. Fluconazole (F8380) and estradiol benzoate (E8430) were acquired from Solarbio (Beijing, China).

### 2.2. Candida Albicans Strain

The *C. albicans* ATCC 64547 used in this study was obtained from the American Type Culture Collection (ATCC; Manassas, VA, USA) and stored in frozen stock with 15% glycerol at −80 °C. Prior to each experiment, the *C. albicans* was seeded into a suspension with sabouraud dextrose agar (SDA) to ensure optimal growth characteristics and purity.

### 2.3. Experimental Animals

144 6-weeks-old Female BALB/c mice (22 ± 2 g) were purchased from Hua Fukang (Beijing, China). All mice were randomly divided into six groups of 24 mice: (1) normal control group (Control, on the day of infection with *C. albicans*, the same vaginal stimulation was administered with PBS simulating the pathogen, and in subsequent treatment, 0.01% Tween-80, which is the PAE solvent treatment was achieved parallel to the infection group and treatment group); (2) *C. albicans* infected mice and 0.01% Tween-80 treatment group (Ca); (3–6) *C. albicans* infected mice and daily vaginal injection with 0.77, 1.54, 3.08 mg/kg PAE, respectively, and 20 mg/kg fluconazole (FCZ), treatment group was used as a therapeutic effect counterpoint to PAE.

Before the start of treatment, all mice were provided with water and food in the IVC system for one week and adapted under regular conditions (a stable temperature of 22–24 °C, a relative humidity of 60%, and a 12/12-h light/dark cycle). All experiments complied with the ethical guidelines for the care and use of laboratory animals of the Chinese Ministry of Science and Technology and the ethical guidelines for the Institutional Animal Care and Use Committee of Jiangsu Normal University.

### 2.4. Construction of the Animal Model of VVC

For appropriate referencing, the model of VVC is made in accordance to previous reports [27]. The estradiol benzoate was dissolved in sesame oil and sonicated to aid with dissolving. PAE was diluted in 0.01% Tween-80, an effective non-ionic surfactant, in order to prepare the PAE as microemulsion. Microemulsion has been reported to be an effective solubilizing carrier for drugs as well as a protective medium for drug degradation, hydrolysis and oxidation to increase drug stability and enhance solubility, release rate, osmosis and absorption [28,29]. Fluconazole (FCZ) powder was prepared in sterile saline.

For modeling, mice in the Ca group and the treatment group were injected with 0.2 mg estradiol benzoate every other day to promote pseudoestrus and *C. albicans* infection, a total of three times a week. Control group mice were injected with sesame oil, which is the solvent of estradiol benzoate. On the day of *C. albicans* infection, 1 μg atropine sulfate was first administered and injected to calm all the groups’ mice. Ten minutes later, all mice were anesthetized with 200 mg/mL urethane. Afterwards, Ca, PAE and FCZ treatment group mice were inoculated into the vaginal lumen with 20 µL of 6 × 10^8^ cells/mL *C. albicans* spores. Control mice were given the same stimulus with the PBS during this period. Afterwards, the PAE and FCZ treatment groups were treated with PAE or FCZ in the morning and afternoon for five days. The Control group and Ca group were treated with 0.01% Tween-80, which is the PAE solvent for five days. At the end of the study, the mice were sacrificed, and vaginal tissue was collected and stored at −80 °C.

### 2.5. Measurement of Intracellular ROS in Vaginal Tissue

Measurement of ROS was performed as described previously with a slight modification [14,30]. Freshly excised vaginal tissue was homogenated by a homogenizer (Bioprep-24, Guangzhou Hangxin Scientific Instrument Co., LTD, Guangzhou, China) and then digested enzymatically for 2 h by 0.1% type 3 collagenase (Worthington Biochemical Co., Lakewood, NJ, USA) at 37 °C with a gentle shaking water bath. Afterward, the isolated cell clusters were centrifuged at 1500× *g* for 8 min and resuspended by HBSS solution (Gibco, Grand Island, NY, USA). Finally, cells were incubated with DCFH-DA (Sigma, St. Louis, MO, USA) at 37 °C for 30 min and filtered with flow tube before testing. The intracellular ROS were detected by an Accuri C6 flow cytometer (BD Biosciences, San Jose, CA, USA).

### 2.6. The Determination of Antioxidant Enzyme Activity in Vaginal Tissue

The MDA, SOD, CAT and GSH kit were purchased from Solarbio Science and Technology Co., Ltd. (BC0025, BC0175, BC0205, BC1175; Beijing, China) and the GSH-PX kit was acquired from Nanjing Jiancheng Bioengineering Institute (A005-1, Nanjing, China). The frozen vaginal tissues were homogenized by a homogenizer (Bioprep-24, Guangzhou Hangxin Scientific Instrument Co., LTD, Guangzhou, China) at 4 °C using tissue grinding bead and buffers as described by commercial kits for respective enzymes. The enzyme activities were tested according to the manufacturer’s instructions. The OD value was measured with a chemiluminescent/UV spectrophotometer (Thermo Fisher, Waltham, MA, USA).

### 2.7. Western Blot Analysis

Western blot analysis was performed following standard procedures. Accordingly, total protein was extracted using a protein extraction kit (BC3711, Solarbio Science and Technology Co., Ltd., Beijing, China). Cytoplasmic protein and nuclear protein were extracted according to the instructions of the nuclear protein extraction kit (R0050, Solarbio Science and Technology Co., Ltd., Beijing, China). Mitochondrial protein extraction was carried out according to the instructions of the cytoplasmic and mitochondrial protein extraction kit (C500051-0050, Sangon Biotechnology Co., Ltd., Shanghai, China). Protein extracts were boiled at 99 °C for 8 min and concentrations were quantified by BCA Protein Assay Kit (PC0020, Solarbio, Beijing, China). Proteins (40 μg) were separated by SDS-PAGE and transferred to polyvinylidene fluoride (PVDF) membranes (Merck Millipore, Billerica, MA, USA). Then, PVDF membranes were blocked for 1 h with 5% non-fat milk (*m/v*) dissolved in Tris-buffered saline containing 0.1% Tween-20 (TBST) and then washed with TBST. Blocked PVDF membranes were incubated with the following primary antibodies at 4 °C overnight: Rabbit anti-PARP (CST, Danvers, MA, USA), Rabbit anti-caspase-9 (CST, Danvers, MA, USA), Rabbit anti-caspase-3 (CST, Danvers, MA, USA), Rabbit anti-caspase-7 (CST, Danvers, MA, USA), Mouse anti-caspase-1 (CST, Danvers, MA, USA), Rabbit anti-Bcl-2 (CST, Danvers, MA, USA), Rabbit anti-Bax (CST, Danvers, MA, USA), Rabbit anti-HO-1 (Abways, Shanghai, China), Rabbit anti-NOX-2 (Bioss, Beijing, China), Mouse anti-Keap1 (Bioworld, Nanjing, China), Rabbit anti-Nrf2 (Bioworld, Nanjing, China), Mouse anti-Asc (Santa Cruz, Santa Cruz, CA, USA), Goat anti-NLRP3 (Abcam, Cambridge, MA, USA). The next day, after washing with TBST, the membranes were incubated with secondary antibodies: horseradish peroxidase- (HRP-) linked Goat anti-rabbit (CST, Danvers, MA, USA), Donkey anti-goat (Abcam, Cambridge, MA, USA) or Rabbit anti-mouse (CST, Danvers, MA, USA) for 1 h at room temperature on a shaker. The expression levels of target proteins were detected using Western Blot chemiluminescence reagents (GE Healthcare, Little Chalfont, Buckinghamshire, UK) and analyzed using the Image J software.

### 2.8. Immunofluorescence Staining

Vaginal tissues were sectioned and stored at −80 °C. To prepare the sectioned tissue for staining, after drying at 37 °C for 2 h, the slides were boiled in 0.1 mol/L sodium citrate buffer (containing 0.1% Tween-20) for 10 min to expose the antigen binding site. The slides were then treated with 0.2% Triton X-100 for 10–20 min, and rinsed with PBS. A total of 5% BSA was used to block the slides at room temperature for 1–2 h. Goat anti-NLRP3 (Abcam, Cambridge, MA, USA) was used as the primary antibody, and the next day, Donkey anti-Goat Alexa Fluor 488 (Abcam, Cambridge, MA, USA) was applied as the secondary antibody. The sections were incubated at room temperature for 40 min-1.5 h and then washed with PBST to remove unbound antibodies. Finally, the slides were stained with DAPI (C0065, Solarbio, Beijing, China) for 30 min in the dark. The sections were examined under a Leica microscope (Leica, Wetzlar, Germany).

### 2.9. Apoptosis Detection

TUNEL (terminal deoxynucleotidyl transferase dUTP nick-end labeling) assay, along with DAPI staining, was employed to evaluate the nuclear condensation and DNA strand breaks. Assays were performed according to the manufacturer’s instructions using an in-situ cell death detection kit (T2190, Solarbio, Beijing, China). Immunofluorescent images were observed under a fluorescence microscope (Leica, Wetzlar, Germany).

### 2.10. ELISA Measurement of IL-1β

The vaginal tissues were homogenized by lysis buffer including protease inhibitor and red blood cell lysate according to the manufacturer’s instructions, followed by centrifugation at 5000× *g* for 10 min. The IL-1β levels in the homogenate were detected using an enzyme-linked immunosorbent assay kit (E-EL-M0037c, Elabscience, Wuhan, China). The OD value was measured with an enzyme-labeled instrument (Thermo Fisher, Waltham, MA, USA).

### 2.11. Statistical Analyses

Statistical analysis and graphing were conducted using the software GraphPad Prism 8.3.0. Data were all shown as mean ± SD. The normality of the continuous variables was evaluated using the Shapiro–Wilk test. The statistical significance between Ca and Control, 0.77 mg/kg, 1.54 mg/kg and 3.08 mg/kg mice was assessed multiple times using the unpaired nonparametric two-tailed Mann–Whitney U test, respectively, with a *p* < 0.05, considered statistically significant, which was used for all statistical analyses. Statistical significance was set at *p* < 0.05 for all tests.

## 3. Results

### 3.1. PAE Reduced Excessive ROS in VVC Mice

**Figure 1:**
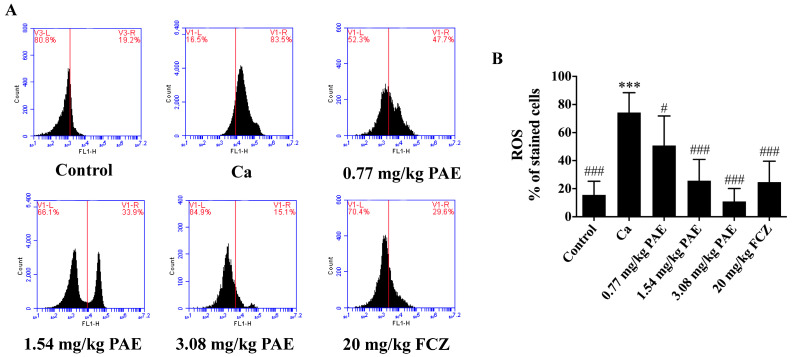
*C. albicans* induced the accumulation of ROS in the vaginal tissues of VVC mice. (**A**) Representative flow cytometry image showing the content of ROS after *C. albicans* and PAE treatment in mice. (**B**) The corresponding statistical analysis of ROS (*n* = 10, *** *p* < 0.001 compared to the Control; # *p <* 0.05 and ### *p* < 0.001 compared to the Ca).

Our previous research has indicated that PAE inhibited the vaginal inflammation afflicted by *C. albicans*. ROS have been regarded as a marker of oxidative stress and an inducer of cell damage or inflammation [13,31]. Therefore, ROS levels in the vaginal tissues of mice were examined. As shown in Figure 1, intravaginal ROS levels in VVC mice increased four times compared to that of the control group. On treating with PAE, the ROS levels in the vaginal tissues gradually decreased, indicating a dose dependency in a statistically significant manner. The 3.08 mg/kg PAE treatment group showed significant differences in ROS levels compared to that of the Ca group and no significant differences compared to that of the control group. In addition, the ROS content in the 3.08 mg/kg PAE treatment group was slightly lower than that in the FCZ treatment group.

### 3.2. PAE Increased Vaginal Antioxidant Activities in VVC Mice

**Figure 2:**
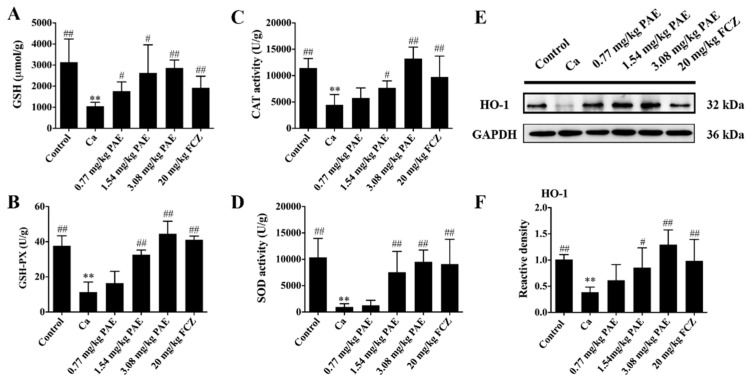
PAE reversed the activity of antioxidant enzymes in VVC mice. (**A**) PAE increased the activity of GSH, GSH-PX (**B**), CAT (**C**) and SOD (**D**), which were down-regulated by *C. albicans* in the vagina. (**E**,**F**) Representative Western blot image showing the HO-1 after *C. albicans* and PAE treatment in mice, with the corresponding gray value analysis (*n* = 6, ** *p* < 0.01 compared to Control; # *p* < 0.05 and ## *p* < 0.01 compared to Ca).

Glutathione (GSH) participates in many cellular reactions and effectively removes free radicals and other ROS (such as hydroxyl radicals, lipid peroxy radicals, peroxy nitrites, H_2_O_2_) through direct or indirect enzymatic reactions [32]. Furthermore, Glutathione peroxidase (GSH-Px) is an important H_2_O_2_ degrading enzyme in mammals, which can catalyze reduced glutathione into oxidized glutathione to protect cells from ROS damage thence, maintain cell structure and function [33]. As shown in Figure 2A,B, compared with mice in the control group, the GSH content or GSH-Px activity in Ca group mice were decreased in a statistically significant manner. After treating with increasing PAE concentration, the GSH content or GSH-Px activity returned to normal levels, which indicated that PAE restored GSH-Px activity and GSH content in a statistically significant manner to remove excess ROS in the vaginal tissues of VVC mice.

Protein catalysts can greatly increase the conversion rate of H_2_O_2_ to O_2_ and H_2_O. CAT is a protein catalyst with substrate specificity. CAT can quickly catalyze the decomposition of H_2_O_2_ into harmless or less toxic substances [34,35]. As shown in Figure 2C, CAT activity in the Ca group mice was reduced by *C. albicans* in the vagina. CAT activity in VVC mice was increased in a statistically significant manner when treated with increasing concentrations of PAE. In addition, CAT activity in the vaginal tissues in the 3.08 mg/kg PAE treatment group showed a significant difference compared to that of the Ca group, higher than the FCZ treatment group, and was returned to the normal level. SOD is another important antioxidant enzyme, which can conveniently remove excessive ROS and repair damaged cells invaded by oxidative free radicals [36]. This study demonstrated that PAE treatment in a statistically significant manner increased SOD activity in VVC mice and the SOD activity of the 3.08 mg/kg PAE treatment mice group was returned to Control group level (Figure 2D). Finally, heme oxygenase 1 (HO-1) is an oxidative stress inducible type within the HO system. This is a 32 kDa stress protein that breaks down heme into bilirubin, free iron, and carbon monoxide. HO-1 is highly sensitive to oxidative stress and has been shown to induce effects in the brain and other tissues in a variety of disease and trauma models [37]. As shown in Figure 2E,F, the expression of HO-1 in the Ca group was inhibited in a statistically significant manner compared to that of the control group. Upon PAE treatment, HO-1 expression in the vaginal tissues was restored. In summary, PAE treatment improved the activities of antioxidant enzymes in the vaginal tissues, eliminated excessive ROS free radicals, protected the vaginal tissues from oxidative damage, and maintained the normal tissue structure and function.

### 3.3. PAE Reduced Vaginal NOX2 Expression and Lipid Peroxide Content

**Figure 3:**
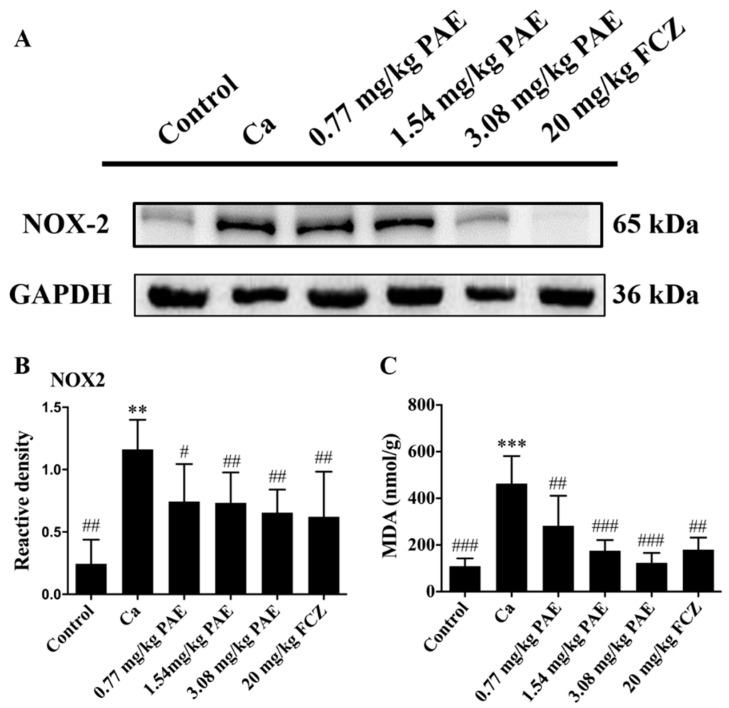
The ROS levels were increased through NOX2 in VVC mice. (**A**,**B**) Western blot and relative density analysis of NOX2 expression in VVC mice (*n* = 6, ** *p* < 0.01 compared to Control; # *p* < 0.05 and ## *p* < 0.01 compared to Ca). (**C**) The content of MDA was increased in *C. albicans* infected mice, which was down-regulated by different concentrations of PAE (*n* = 8, *** *p* < 0.001 compared to Control; ## *p* < 0.01 and ### *p* < 0.001 compared to Ca).

NADPH oxidase (NOXs) is one of the main sources of reactive oxygen species in the vascular region, leading to vascular damage and a variety of cardiovascular diseases. Among the NOX subtypes, nicotinamide adenine dinucleotide phosphate oxidase 2 (NOX2) is widely expressed in a variety of cell types, such as cardiomyocytes, endothelial cells, and vascular smooth muscle cells. ROS production could be induced by activating phagocytic NOX2 complexes, a process commonly referred to as oxidative burst [38]. Therefore, the correlation between the accumulation of ROS in the vaginal tissues of VVC mice and the up-regulated expression of NOX2 was investigated. As shown in Figure 3A,B, *C. albicans* infection increased the expression of NOX2 in the vaginal tissues, which can lead to oxidative damage, while 3.08 mg/mL PAE inhibited the expression of NOX2 protein in a statistically significant manner. Additionally, malondialdehyde (MDA) is the main product of polyunsaturated fatty acid peroxidation. This aldehyde is a highly toxic molecule and is considered a marker of lipid peroxidation [39]. As shown in Figure 3C, MDA in the vaginal tissues of the Ca group was higher in a statistically significant manner than that in the control group. PAE treatment reduced the intravaginal MDA content of VVC mice to lower than that of Ca group mice. Therefore, PAE reduced vaginal NOX2 expression and MDA content induced by *C. albicans*.

### 3.4. PAE Enhanced Vaginal Nrf2 Activity in VVC Mice

**Figure 4:**
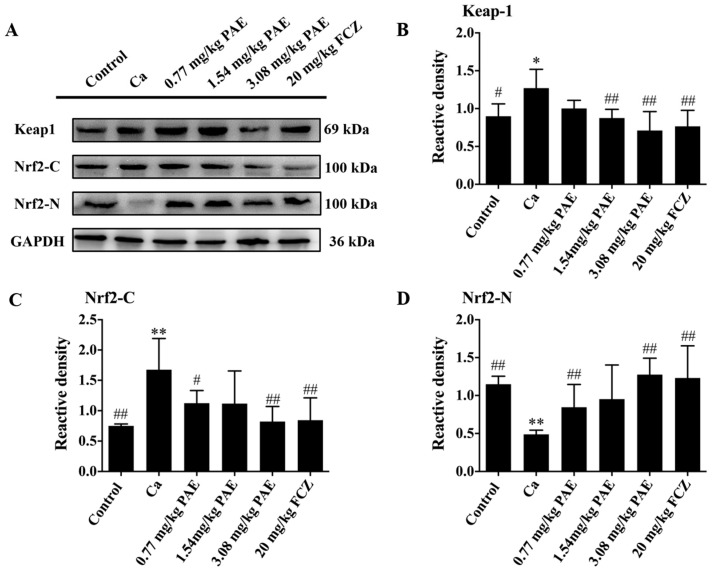
PAE exerted antioxidant effect via Nrf2 in VVC mice. (**A**–**D**) Representative Western blot image showing the Keap1, Nrf2 in cytoplasm and Nrf2 in nucleus after *C. albicans* and different concentrations of PAE treatment in mice, with the corresponding gray value analysis (*n* = 6, * *p* < 0.05 and ** *p* < 0.01, compared to Control; # *p* < 0.05 and ## *p* < 0.01 compared to Ca).

The current study has shown that VVC led to increases in the intravaginal ROS and MDA content, while the antioxidant enzyme activities decreased obviously. Therefore, the protective effects of PAE through the mechanism of antioxidation were investigated. Nrf2 is one of the important bZIP transcription factors, which could form heterodimers with other bZIPs to regulate oxidative stress response [40]. This can be transferred from the cytoplasm to the nucleus to activate the target gene’s promoter regions of the antioxidant response element (ARE), which regulates the genes expression of tissue oxidative stress, and removes the excessive free radicals. In addition, kelch-like ech-associated protein 1(Keap1) is an Nrf2 inhibitor that can bind onto intracellular Nrf2, retain Nrf2 in the cytoplasm, and conduct targeted ubiquitination degradation of Nrf2 in the cytoplasm [41]. As shown in Figure 4A–D, the expression of the Nrf2 binding protein Keap1 in the vaginal tissues of VVC mice was increased. Low expression of nuclear Nrf2 was detected in the Ca group, while the expression of nuclear Nrf2 was recovered to a moderately normal level in the 3.08 mg/kg PAE treatment group. Therefore, PAE exerted its function by targeting Keap1, promoting the Nrf2 transferred to the nuclear Nrf2 to restore the antioxidant function, and by protecting the vaginal tissues of VVC mice.

### 3.5. PAE Inhibited Vagina Apoptosis Induced by C. albicans Invasion

**Figure 5:**
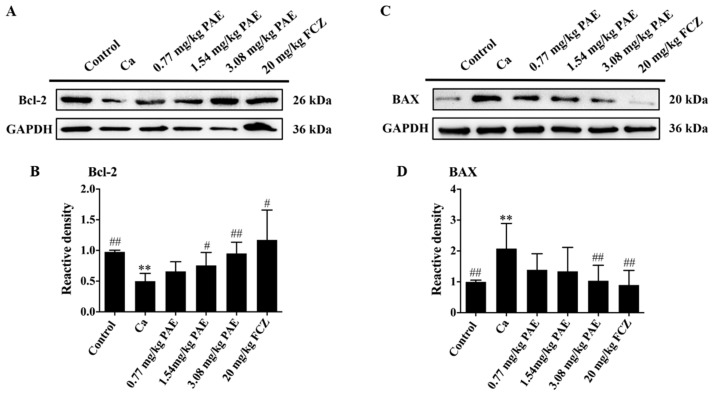
PAE increased the expression of anti-apoptotic protein Bcl-2 and decreased the expression of pro-apoptotic protein BAX in VVC mice. (**A**–**D**) Representative Western blot image showing the Bcl-2 and BAX after *C. albicans* and different concentrations of PAE treatment in mice, with the corresponding gray value analysis (*n* = 6, ** *p* < 0.01 compared to Control; # *p* < 0.05 and ## *p* < 0.01 compared to Ca).

One of the most fascinating discoveries in cell biology is that mitochondria have largely represented the convergence point of many apoptosis-inducing signals in mammalian cells [42]. ROS has long been considered to be one of the main factors that disrupt mitochondrial function and induce apoptosis [43]. BAX is a vital component of apoptosis induced by mitochondrial stress. In addition, under various apoptotic stimulation Bcl-2 plays an anti-apoptotic role by inhibiting the release of mitochondrial cytochrome C [44,45]. Therefore, the presence and level of mitochondrial proteins in VVC mice were analyzed. As shown in Figure 5A–D, *C. albicans* infection inhibited the expression of Bcl-2 and induced overexpression of BAX in a statistically significant manner in the vaginal tissues of VVC mice. PAE treatments reversed such changes and these results indicated that PAE could ameliorate vaginal mitochondrial damage in VVC mice.

**Figure 6:**
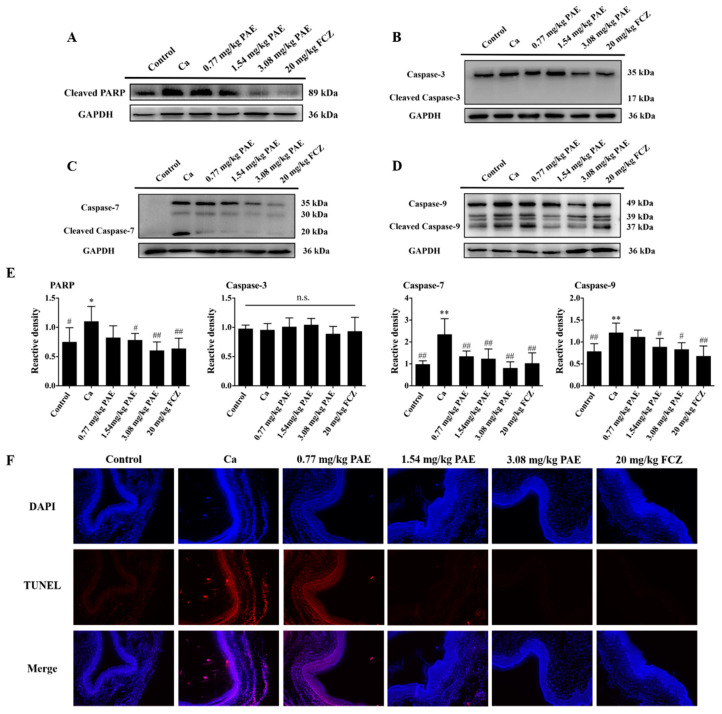
PAE inhibited the apoptosis induced by *C. albicans* in mice vaginas through caspase 9-caspase 7-PARP pathway. (**A**–**E**) Representative Western blot image showing the cleaved-PARP, caspase-3, caspase-7 and caspase-9 after *C. albicans* and different concentrations of PAE treatment in mice, with the corresponding gray value analysis (*n* = 6, * *p* < 0.05 and ** *p* < 0.01 compared to Control; # *p* < 0.05 and ## *p* < 0.01 compared to Ca, n.s. means no significant). (**F**) The apoptosis in the VVC mice were detected by TUNEL and DAPI staining to show apoptosis cells (red) and the nucleus (blue).

Poly ADP-ribose polymerase (PARP) maintains cell viability and PARP cleavage, in vivo, promotes the disintegration of cells and triggers apoptosis, which is an important marker of apoptosis [46,47]. Hence, the expression of PARP in the vaginal tissues was examined. As shown in Figure 6A, the expression of PARP was increased in a statistically significant manner in the Ca group, and 3.08 mg/kg PAE treatment restored the expression of PARP to the Control group level.

Caspase is another important regulatory family of apoptosis. When stimulated by an apoptotic inducer, Caspase 9 is further processed, and the lysed Caspase 9, as the initiator of apoptosis, further activates the apoptotic executor Caspase 3 or Caspase 7, thus triggering apoptosis [48]. As shown in Figure 6B–D, the protein expression of Caspase 9 in the Ca group was increased in a statistically significant manner, and 3.08 mg/kg PAE treatment was able to reduce the expression of Caspase 9 nearly to the Control group level in a statistically significant manner. The changes in expression of Caspase 9 is consistent with that of Caspase 7 in the vaginal tissues. Therefore, we speculate that the main executor of apoptosis in the vaginal tissues of VVC mice is Caspase 7. At the same time, no significant differences in the expression of Caspase 3 were observed in this study, which also confirmed that the main executor of apoptosis in the VVC mice is Caspase 7. Finally, TUNEL staining was performed to further analyze the effect of PAE (Figure 6F). There was obvious apoptosis in the vaginal tissues of VVC mice, which was reduced in a statistically significant manner in the PAE treatment groups.

### 3.6. PAE Suppressed the IL-1β Release in VVC Mice

**Figure 7:**
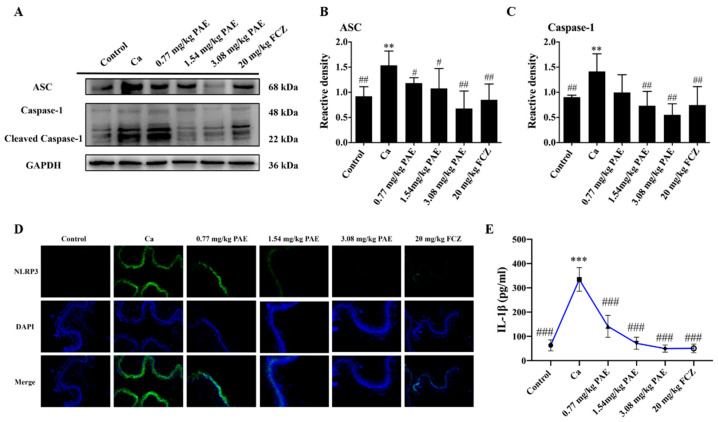
PAE suppressed the release of IL-1β in VVC mice. (**A**–**C**) Representative Western blot image showing the ASC and caspase-1 after *C. albicans* and different concentrations of PAE treatment in mice, with the corresponding gray value analysis (*n* = 6, ** *p* < 0.01 compared to Control; # *p* < 0.05 and ## *p* < 0.01 compared to Ca). (**D**) Immunofluorescence analysis of the expression of NLRP3 to visualize the NLRP3 cells (green) and nucleus (blue), respectively. (**E**) Quantitation of the IL-1β release in VVC mice after being treated by PAE (*n* = 8, *** *p* < 0.001 compared to Control; ### *p* < 0.001 compared to Ca).

Our previous study has demonstrated that PAE decreased inflammatory cytokines and immune cells in the vagina of VVC mice [13], yet the mechanism is not clear. In this study, it has also been shown that PAE reduced the ROS levels in VVC mice. ROS is one of the key factors in the assembly of NLRP3 inflammasome [49]. Therefore, it was hypothesized that PAE could suppress the IL-1β release to relieve the inflammation in vaginal tissues. As shown in Figure 7A–D, the expressions of ASC, Caspase 1 and NLRP3 in the vaginal tissues of the Ca group were increased, while their VVC-induced expressions were alleviated by PAE treatments. In addition, *C. albicans* invasion mediated enhanced expression of IL-1β, and 3.08 mg/kg PAE treatment brought it back to a normal level in a statistically significant manner (Figure 7E). Therefore, PAE inhibited the IL-1β release and reduced the inflammatory response mediated by *C. albicans* infection in the vaginal tissues of VVC mice.

## 4. Discussion

ROS are important physiological signal molecules formed from a redox donor by one-electron transfer to molecular oxygen (O_2_) in the body [50]. If ROS were allowed to accumulate excessively, it could damage mitochondrial proteins, organelle membranes, and DNA, which would then lead to cell senescence, malignant transformation and apoptosis [51]. In this study, we have shown that PAE can inhibit ROS accumulation mediated by *C. albicans* infection in the vaginal tissues, reduce oxidative damage, apoptosis and inflammatory responses. As a new type of antifungal agent, not only can PAE inhibit pathogenic microorganism proliferation, it can also protect the host from tissue damage mediated by excessive ROS, which may both contribute to the therapeutic effects of PAE. Cells are equipped with multiple antioxidant systems that limit ROS-induced oxidative damage. Strict control of ROS content in vivo is necessary to improve the body’s antioxidant capacity and prevent pathological mutations in host cells [52]. In this study, we found that ROS homeostasis in mouse vaginal tissues was disrupted by *C. albicans*, and a large amount of ROS were detected. We hypothesized that an excessive amount of ROS activate a series of stress reactions in the vaginal cells of VVC mice and cause severe damage to tissues. We also hypothesized that an excessive amount of ROS reduce the efficacy of antifungal agents, resulting in excess ROS residue in the body tissue and this interferes with the appropriate response of the host immune system to *C. albicans*. Relevant studies have also indicated that the excess ROS around the tumor will also hinder the potency of anti-tumor drugs, and this is consistent with our conjecture [53,54]. Unfortunately, CAR T cells are particularly susceptible to adverse inflammatory conditions, and relevant studies have indicated that the regulation of their ROS levels may help maintain their effector function and viability [34]. Antineoplastic drugs are also commonly used as antifungal drugs, and they possess the same targets [55]. Therefore, effective targeting of excessive ROS in the body may further promote the therapeutic effect of PAE on fungal infections. Combo therapy, such as anti-tumor therapy together with antioxidant compounds, may present a promising way to overcome treatment resistance and obtain better outcomes, as preemptive metabolic adaptation may contribute to the overall efficacy of immunotherapy [56].

Excess ROS produced in cells, will be quickly broken down by various antioxidant proteins to form low-toxic or non-toxic substances under normal circumstances [57]. In some animal studies, dietary antioxidants or calorie restrictions, and the increased expression of chemical antioxidants or antioxidant proteins, have been shown to reduce ROS production and extend lifespan [58]. Herein, we also discovered that when the activities of antioxidant enzymes such as SOD, CAT and HO-1 in the vaginal tissues of VVC mice were inhibited, GSH content was reduced in a statistically significant manner. As an antioxidant and antimicrobial agent, PAE functions by inhibiting pathogenic microorganism proliferation and by also improving the antioxidant capacity, thus protecting the vagina from oxidative damage mediated by excessive ROS. Furthermore, in our research, we found that *C. albicans* infection resulted in an increase in the expression of NOX2 protein in the vaginal tissues of VVC mice. NOX2 have been proven to possess multiple functions and it is very important for the maintenance of a physiological condition. NOX2 is recognized as the major ROS-generating enzyme and it is involved in the pathogenesis of a range of diseases, including atherosclerosis, cancer, and neurodegenerative diseases. In addition, NOX2 is also considered to be a host defense agent and an inflammatory agent, which is consistent with our results [13,59]. At the same time, NOX2 has been reported to specifically activate signaling pathways, such as Nrf2, thus becoming an important therapeutic target for chronic granulomatous and Crohn’s disease, in which severe bacterial or fungal infections and excessive inflammation are the main characteristics. More broadly, Crohn’s disease may be a disease associated with excessive inflammation and injury, which is consistent with our research [59,60]. We found that the expression of Keap1 in the Ca group mice was increased in a statistically significant manner, and this implies that a large amount of Nuclear factor E2 related factor 2 (Nrf2) is trapped in its cytoplasm thence causing the inhibition of the oxidative stress response in VVC mice with a tendency of creating oxidative damage in the vaginal cells. Upon treatments with different concentrations of PAE, the expression of Keap1 in the vaginal tissues decreased to the normal level, as well as the expression of Nrf2 in the nucleus. If excess ROS cannot be cleared on time, it may also induce apoptosis. Our study showed that *C. albicans* infection can lead to overexpression of intravaginal BAX and the inhibition of Bcl-2, thereby activating Caspase 9, which is the initiator of apoptosis. The activated Caspase 9 then cleaved the apoptotic performer Caspase 7 instead of Caspase 3, thereby activating PARP and inducing apoptosis. Similarly, it has been reported that in rectal cancer, SREBP1 also inhibited PARP cleavage mainly by reducing the expression of Caspase 7 instead of Caspase 3, which was consistent with our results [61].

ROS are also considered to be an important marker for activating NLRP3 inflammasome, which trigger inflammation in the body [26]. NLRP3 inflammasome was activated in the vaginal tissues of VVC mice and led to the onset of host inflammation, which is consistent with our previous findings. In previous studies, we found that PAE inhibited the expression of inflammatory cytokines and reduce the content of immune cells in a statistically significant manner in the vaginal tissues, which is consistent with the results of PAE in inhibiting the release of IL-1ꞵ in this study [13]. Furthermore, some studies have also confirmed that PAE inhibited depressive behavior in rats through the NLRP3 pathway and treated colitis by reducing the content of proinflammatory cytokines in the intestine [22,23]. Therefore, PAE, as a natural active ingredient that can effectively inhibit inflammatory responses, may exert its effects via the targeting of NLRP3 inflammasome.

**Figure 8:**
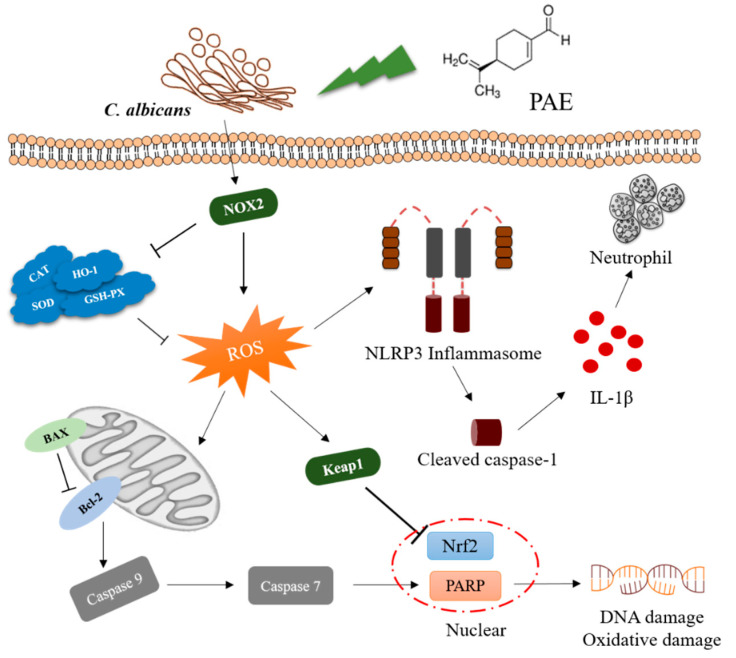
The schematic drawing of the proposed mechanism of PAE against oxidative stress and apoptosis in VVC mice.

Collectively, PAE is not weaker than fluconazole in the treatment of VVC and the proposed mechanism of PAE in ameliorating VVC is shown in Figure 8. This study shows that *C. albicans* infection increased the expression of NOX2 in the vaginal tissues of mice and inhibited the activities of antioxidant enzymes such as SOD, CAT, HO-1 and GSH-Px in a statistically significant manner. Meanwhile, *C. albicans* infection also reduced GSH concentration and increased ROS and MDA content. Furthermore, PAE inhibited Keap1 activity, promoted Nrf2 into the nucleus, and achieved preventative effects against oxidative stress and tissue damage. We also discovered that PAE inhibited the apoptosis and inflammatory response mediated by excessive ROS. Upon PAE treatment, the expression of anti-apoptotic protein Bcl-2 was increased while the expression of pro-apoptotic protein BAX was decreased. PAE inhibited the apoptosis in VVC mice through Caspase 9-Caspase 7 -PARP pathway. Finally, PAE also inhibited the release of IL-1ꞵ to reduce the inflammatory response in VVC mice. By elucidating the mechanism of PAE preventing VVC, this study therefore provides a theoretical basis for the further development of PAE into clinical antifungal drugs.

## 5. Conclusions

The ongoing VVC demands novel and effective therapies to restrict *C. albicans* resistance and lessen tissue damage. Our research expounded that *C. albicans* infection accumulated the excessive ROS and promotes caspase-7 dependent apoptosis in the vaginal tissues of VVC mice. Meanwhile, excessive ROS induces the assemble of NLRP3 inflammasome. More importantly, we found that PAE, a natural monoterpene extracted from *Perilla frutescens*, blocks ROS accumulation by inhibited the expression of NOX2 and up-regulated the activities of antioxidant enzymes in the vaginal tissues. In addition, we indicated that PAE is capable to prevent the cleaved of caspase-7 rather than caspase-3, and the assemble of NLRP3, thus suppressed the inflammatory response and apoptosis to decrease the tissue damage. It has been previously demonstrated that PAE exhibited the low toxicity and has been certified as generally safe as a food additive. Therefore, PAE as an anti-oxidant should be highly emphasized. Clinical trials are essential to evaluate the effect of PAE in patients with VVC will propel its clinical application.

## Data Availability

Data is contained within the article.
